# P-901. Characterizing Changes in Antibiotic Use in the Outpatient Setting – United States, 2019 and 2022

**DOI:** 10.1093/ofid/ofaf695.1107

**Published:** 2026-01-11

**Authors:** Christine Kim, Tessa Schwarze, Lauri A Hicks, Adam Hersh, Sarah Kabbani, Emily McDonald

**Affiliations:** CDC Division of Healthcare Quality Promotion, Atlanta, Georgia; Chenega Enterprises Systems & Solutions, Washington DC, District of Columbia; Centers for Disease Control and Prevention, Atlanta, GA; University of Utah, Salt Lake City, Utah; Centers for Disease Control and Prevention, Atlanta, GA; Centers for Disease Control and Prevention, Atlanta, GA

## Abstract

**Background:**

Unnecessary antibiotic use is common in outpatient settings, particularly for acute respiratory illnesses. Monitoring antibiotic prescribing and appropriateness is important for identifying stewardship priorities and tracking progress toward national goals. We evaluated changes in outpatient antibiotic prescribing practices and estimated proportions of unnecessary prescribing.

**Figure 1.**
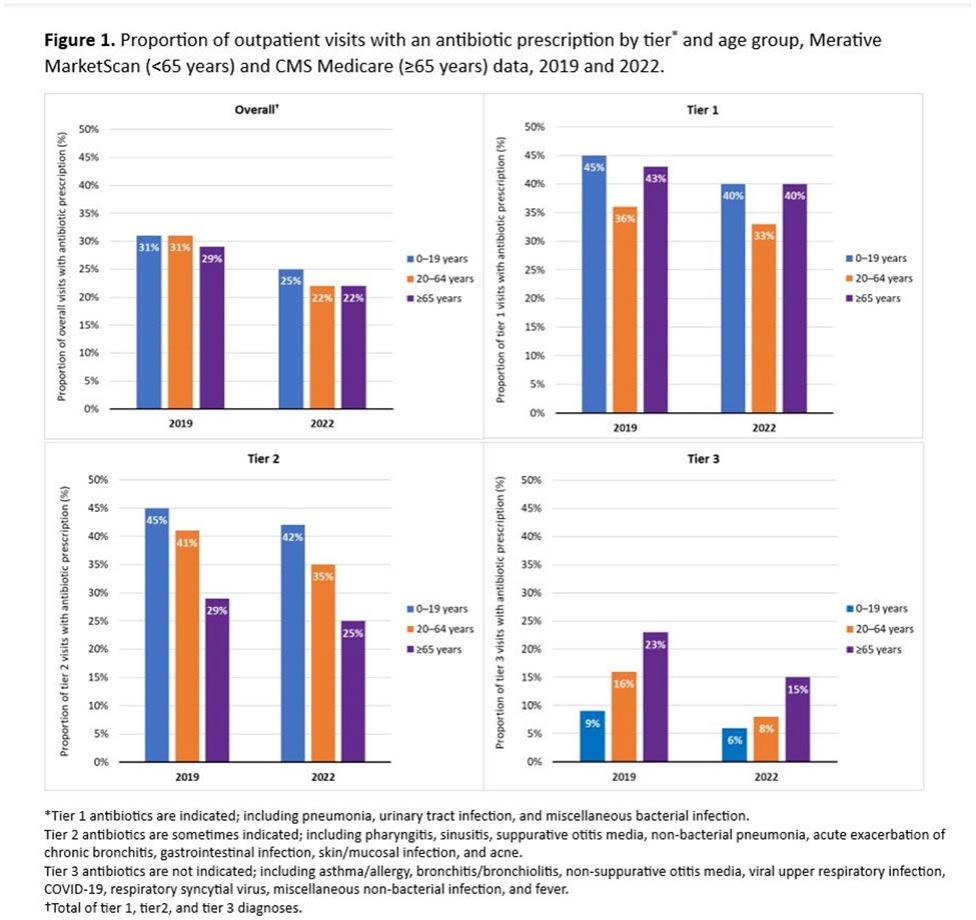
Proportion of outpatient visits with an antibiotic prescription by tier* and age group, Merative MarketScan (<65 years) and CMS Medicare (≥65 years) data, 2019 and 2022. *Tier 1 antibiotics are indicated; including pneumonia, urinary tract infection, and miscellaneous bacterial infection. Tier 2 antibiotics are sometimes indicated; including pharyngitis, sinusitis, suppurative otitis media, non-bacterial pneumonia, acute exacerbation of chronic bronchitis, gastrointestinal infection, skin/mucosal infection, and acne. Tier 3 antibiotics are not indicated; including asthma/allergy, bronchitis/bronchiolitis, non-suppurative otitis media, viral upper respiratory infection, COVID-19, respiratory syncytial virus, miscellaneous non-bacterial infection, and fever. †Total of tier 1, tier2, and tier 3 diagnoses.

**Figure 2.**
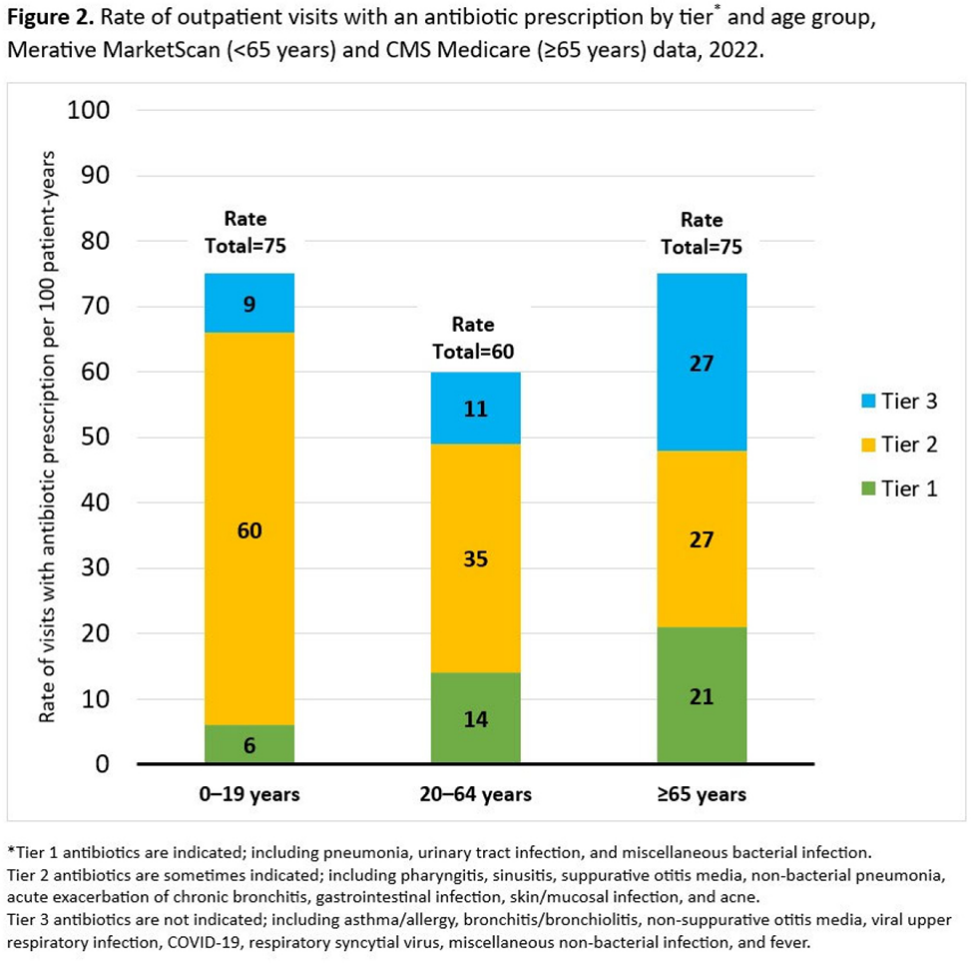
Rate of outpatient visits with an antibiotic prescription by tier* and age group, Merative MarketScan (<65 years) and CMS Medicare (≥65 years) data, 2022. *Tier 1 antibiotics are indicated; including pneumonia, urinary tract infection, and miscellaneous bacterial infection. Tier 2 antibiotics are sometimes indicated; including pharyngitis, sinusitis, suppurative otitis media, non-bacterial pneumonia, acute exacerbation of chronic bronchitis, gastrointestinal infection, skin/mucosal infection, and acne. Tier 3 antibiotics are not indicated; including asthma/allergy, bronchitis/bronchiolitis, non-suppurative otitis media, viral upper respiratory infection, COVID-19, respiratory syncytial virus, miscellaneous non-bacterial infection, and fever.

**Methods:**

We used Merative MarketScan commercial claims (patients < 65 years) and CMS Medicare carrier claims and Part D event files (patients ≥65 years) to identify enrollees with medical and prescription coverage for 2019 and 2022. Enrollees were weighted based on number of months enrolled. A tiered algorithm assigned a single diagnosis to outpatient visits based on most likely indication for antibiotics using ICD-10 codes. Tier 1 included diagnoses where antibiotics are indicated (e.g., pneumonia), tier 2 was diagnoses where antibiotics are sometimes indicated (e.g., sinusitis), and tier 3 was diagnoses where antibiotics are not indicated (e.g., bronchitis). Oral antibiotics dispensed on visit date or within 7 days after were linked. We calculated the proportion and rate (per 100 patient-years) of antibiotic visits by tier and age group, and determined percent change from 2019 to 2022.

**Results:**

The proportion of outpatient visits resulting in an antibiotic was lower in 2022 compared to 2019, regardless of age group or tier (Figure 1). Change in prescribing was greatest for tier 3 conditions, where antibiotic visits decreased 47% for adults ages 20-64 years and 34% for both children ages 0-19 years and adults ages ≥ 65 years. Tier 3 prescribing increased with age and was highest for adults ages ≥ 65 years. During 2022, the highest prescribing rates were for tier 2 conditions, with children ages 0-19 years receiving 60 antibiotics/100 patient-years (Figure 2).

**Conclusion:**

The proportion of antibiotic visits decreased among all age groups in 2022 compared to 2019, with the greatest decline occurring for tier 3 conditions. Opportunities to further reduce unnecessary prescribing for tier 3 conditions exist, particularly among older adults. Stewardship efforts should also prioritize tier 2 conditions, but diagnosis-specific evaluations of appropriate prescribing are needed to guide interventions.

**Disclosures:**

Christine Kim, PhD, Moderna, Inc.: Epidemiologist|Moderna, Inc.: Stocks/Bonds (Public Company)

